# Supplementary material for: Modeling infodemics on a global scale: A 30 countries study using epidemiological and social listening data
Source: PNAS Nexus. 2026 Aug 14;5(9):pgag274. doi: 10.1093/pnasnexus/pgag274 (PMC13525379; doi:10.1093/pnasnexus/pgag274)
Supplement: Supplementary file 1 [file pgag274_supplementary_data.pdf]

Modelling Infodemics on a Global Scale: A 30 Countries Study using Epidemiological and Social Listening Data

Supplementary Material

Edoardo Loru[1], Marco Delmastro[2, 3], Francesco Gesualdo[4], and Matteo Cinelli[5, *]

[1]Sapienza University of Rome, Department of Computer, Control and Management Engineering, Rome, Italy
[2]Ca' Foscari University of Venice, Department of Environmental Sciences, Informatics and Statistics, Venice, Italy
[3]Centro Ricerche Enrico Fermi, Rome, Italy
[4]University of Pisa, Department of Translational Research and New Technologies in Medicine and Surgery, Pisa, Italy
[5]Sapienza University of Rome, Department of Computer Science, Rome, Italy
[*]matteo.cinelli@uniroma1.it

Coupling between epidemic and infodemic using cross-correlation

In order to quantitatively estimate the level of agreement between the number of new documents and the number of new cases, that is the infodemic and epidemic phenomena, we compute the cross-correlation function (CCF) of the two time series, by displacing the latter of $h = 0, \pm 1, \pm 2, \ldots, \pm 25$ days. In this analysis, correlation for negative (positive) values of h indicates that the number of documents precedes (follows) the number of cases, whereas a high correlation for $h = 0$ suggests that the two variables are strongly coupled. In Fig S1, we observe that almost all countries exhibit a positive correlation across all the considered lags, suggesting that the number of new documents and the number of new cases are positively coupled overall. However, we note that not all CCF's display the same profile. For instance, the curves for India, Indonesia, and Senegal are symmetrical around $h = 0$, meaning that the highest correlation is found when the two time series are not displaced with respect to each other. On the other hand, countries such as Angola, the UK, and Spain show a generally decreasing CCF: correlation is highest when the number of cases is shifted back in time, meaning that the number of documents appears to lead the number of cases. Note that the behaviour observed in the case of countries such as France and Switzerland can be interpreted as a specification of this decreasing trend towards negative values. Although not as common, the opposite behaviour (increasing CCF) can also be observed (e.g., for Nicaragua and Yemen). For a final visual inspection, in Fig S2 we highlight how the cross-correlation function for some countries relates to their respective number of documents and number of cases over time.

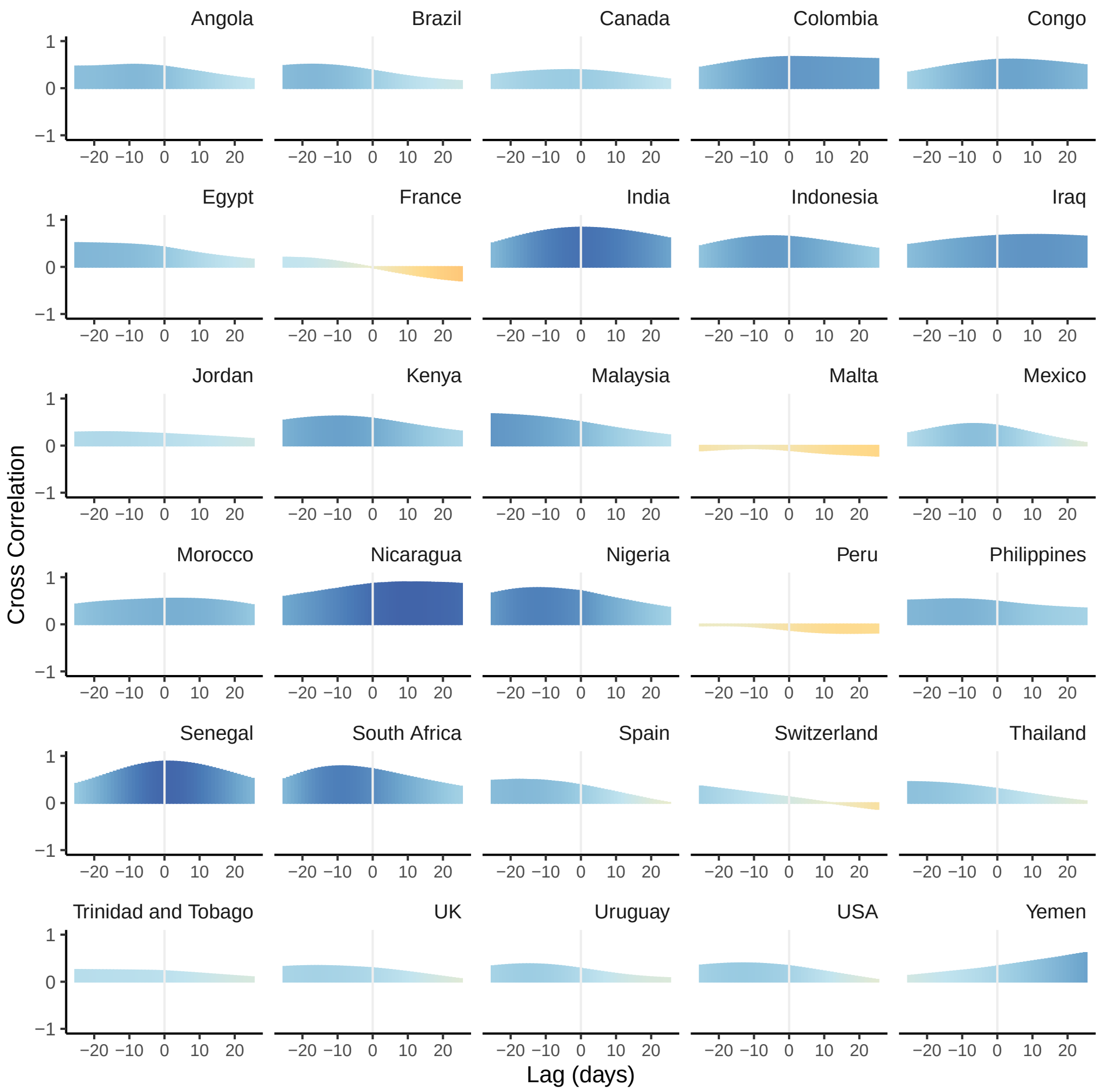

Figure S1: **Cross-correlation between number of new cases and number of new documents.** On the x-axis, how many days the number of documents has been shifted by, with positive (negative) values indicating that the documents time series has been moved backwards (forwards); on the y-axis, the sample correlation between the two time series at the given lag.

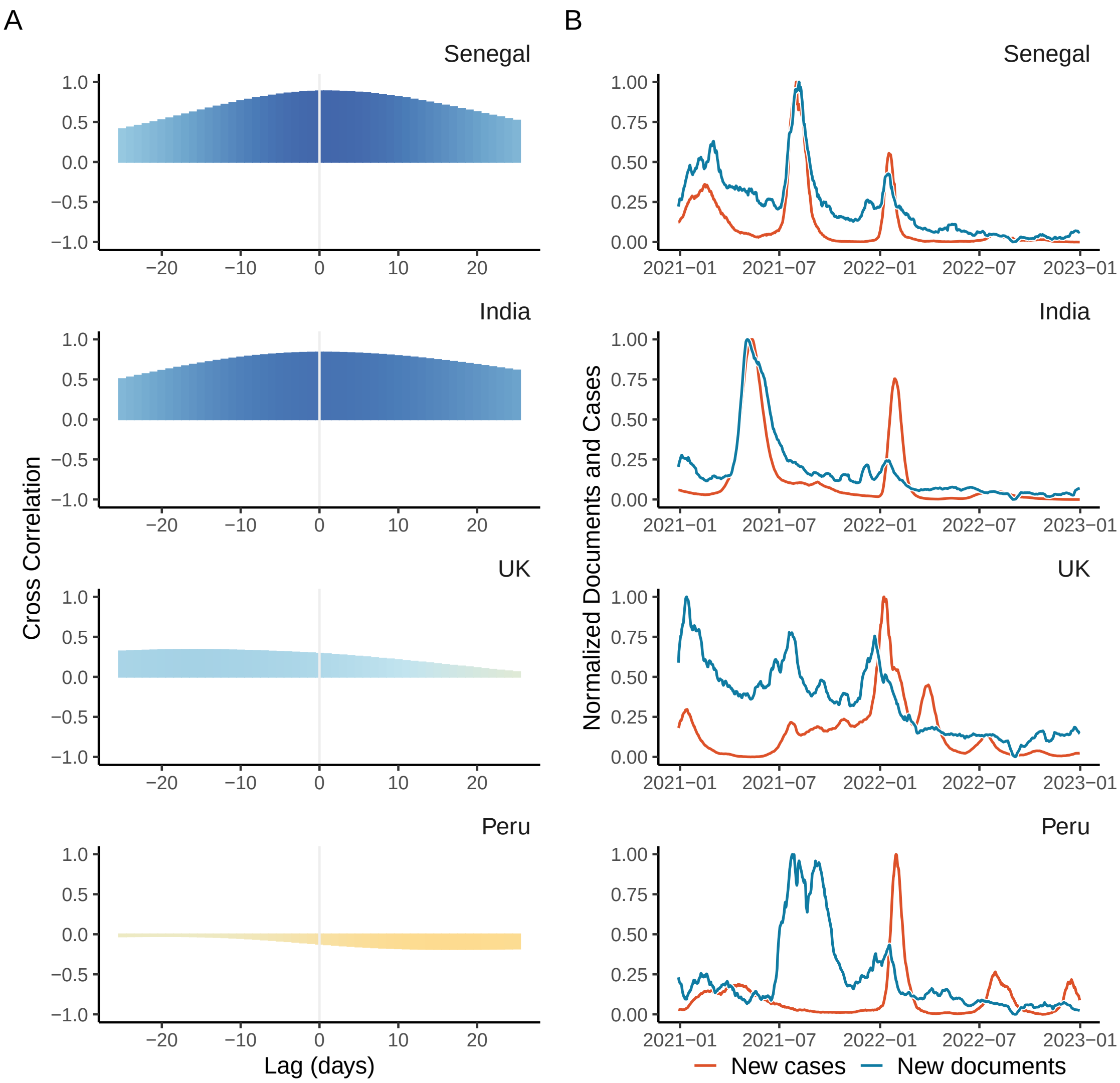

Figure S2: **Cross-correlation between the number of new cases and the number of new documents.** Four paradigmatic cases related to Senegal, India, UK and Peru are displayed.

Transient dynamics of coupling between epidemic and infodemic

The positive cross-correlation observed in the data represents summary information that is useful to understand the general relationship between the variables over the complete time window. Indeed, cross-correlation does not take into account local variations that are present in the data (see for instance the time series in Fig S2). These variations, if present, may represent a localised (e.g., weekly/bi-weekly) decoupling between the epidemic and infodemic curves. To capture these local variations, we employ the scale-dependent correlation analysis (SDC) [3, 2], a method applied to the time series of both epidemiological and infodemiological variables [1]. The SDC plots can be read as follows: each cell of the grid is coloured according to the Spearman correlation coefficient, with the x and y coordinates corresponding to the position of the two respective time windows of size $s = 70$ days along each of the time series. The main diagonal shows the correlation between paired segments ($h = 0$), while other points refer to the correlation between unpaired segments ($h \neq 0$): in the area above the diagonal the first segment of the new cases time series lags the second segment of the new documents time series, while in the area below the diagonal the opposite happens. More in general, the lag h between the cells' position corresponds to the distance from the diagonal ($h \in \{-21, \ldots, +21\}$ days), thus the value of the shift between two fragments is the distance in pixels from any point to the main diagonal: positive lags $h \in [1, 21]$ of the new cases time series with respect to the new documents one are visualised as shifts on the x-axis, whereas negative lags $h \in [-21, -1]$ (that is positive lags of the new documents time series with respect to the new cases one) are visualised as shifts on the y-axis. For instance, the first line of pixels in Fig S3 compares new documents in the interval $[1, 70]$ with new cases in the same interval, while the first pixel on the right of the diagonal compares new documents in the interval $[1, 70]$ with new cases in the interval $[2, 71]$.

The main message deriving from Fig S3, in which only correlations found to be significant in a non-parametric randomised test ($\alpha = 0.01$) are shown and coloured, is systematic evidence for transient dynamics of correlation towards both positive and negative values witnessed by curves oscillating between blue-coloured and red-coloured dots. Nonetheless, not all countries behave the same: some of them display a relatively negative correlation between the two phenomena (e.g., Spain), whereas others (e.g., the UK) switch between positive, negative or non-significant correlation regimes, thus indicating a fluctuating relevance of one variable with respect to the other. While the peculiarity of these coupling dynamics is substantiated by the correlation values themselves, the interplay between new documents and new cases substantially differs from that displayed by comparing epidemiological variables only. In fact, the SDC plot of the number of new cases and new deaths displays, as expected, largely positive correlations (see Fig S4).

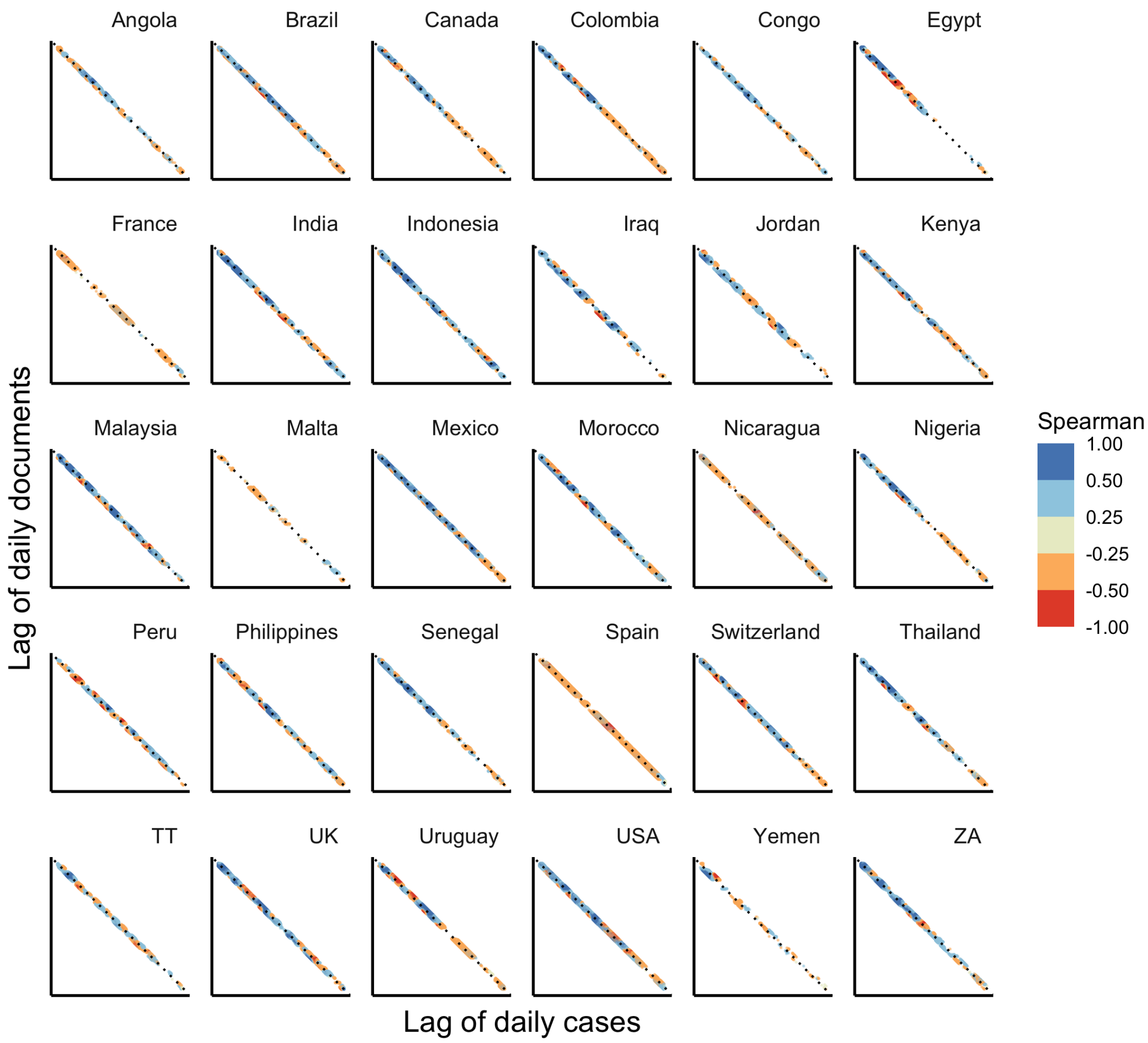

Figure S3: **Scale Dependent Correlation between the number of new daily cases and of new daily documents.** For all time windows of $s = 70$ days of the two time series, the Spearman correlation is computed and reported on the figure if significant ($p < 0.01$); on the main diagonal, the correlation between paired windows is reported, whereas off-diagonal the windows are shifted with respect to each other. Trinidad and Tobago (TT) and South Africa (ZA) are labelled with their country code rather than their name for visualization purposes.

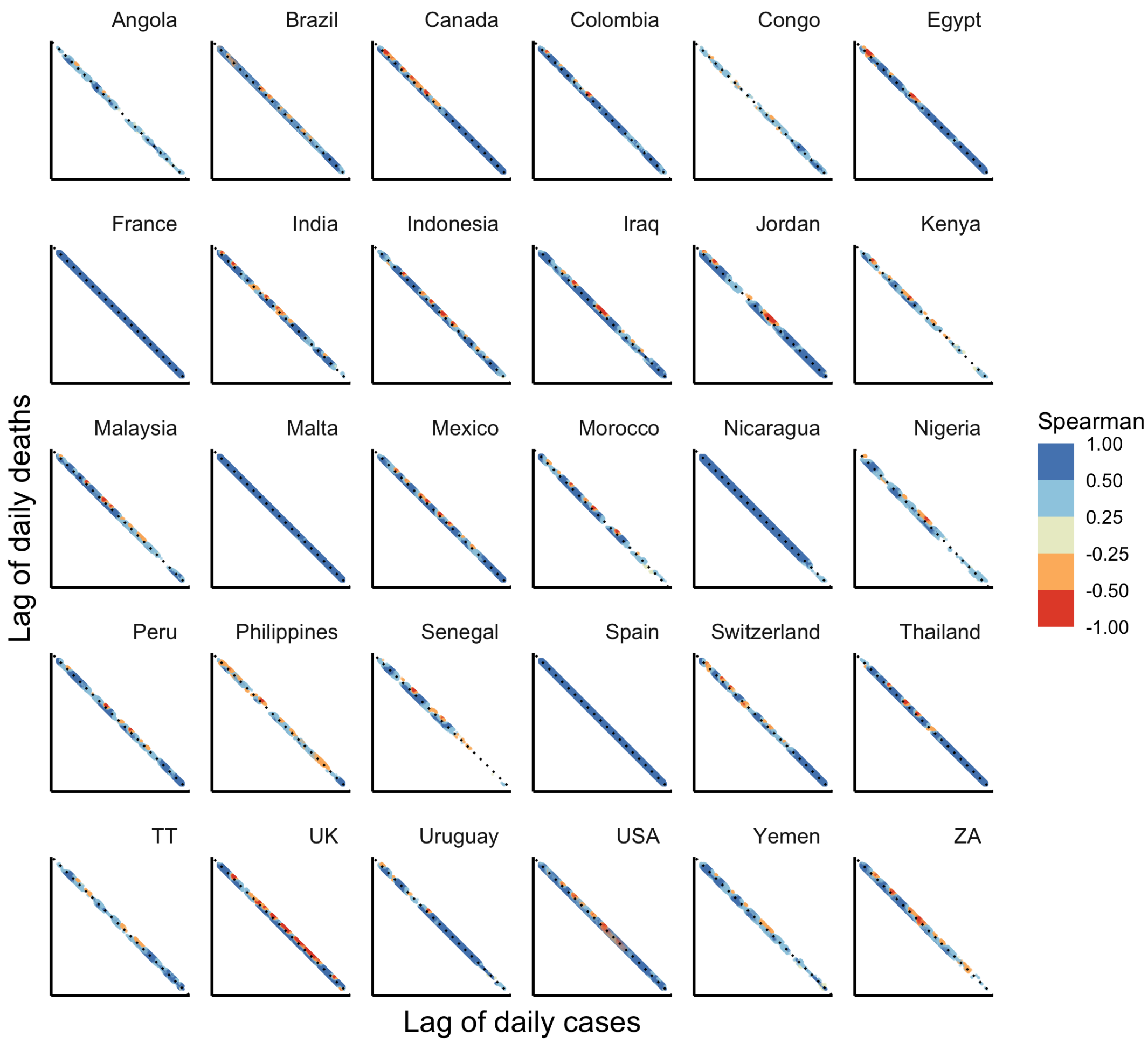

Figure S4: **Scale Dependent Correlation between the number of new daily cases and of new daily deaths.** For all time windows of $s = 70$ days of the two time series, the Spearman correlation is computed and reported on the figure if significant ($p < 0.01$); on the main diagonal, the correlation between paired windows is reported, whereas on the off-diagonal the windows are shifted with respect to each other. Trinidad and Tobago (TT) and South Africa (ZA) are labelled with their country code rather than their name for visualisation purposes.

Robustness checks on regression models

Table S1: **Fixed-effect panel regression with robust standard errors**. As the data provided by Google Trends are weekly, unlike other models this regression only takes into account dummy variables for the season of the year.

	Google Trends		
	(2a)	(2b)	(2c)
New cases	0.150***		0.055*
	(0.025)		(0.024)
New cases (neighbours)	0.132***		−0.123***
	(0.029)		(0.032)
New deaths		0.161***	0.101***
		(0.027)	(0.028)
New deaths (neighbours)		0.287***	0.405***
		(0.025)	(0.033)
Observations	6,000	6,000	6,000
R^2	0.440	0.568	0.582
Adjusted R^2	0.437	0.566	0.580

*p<0.1; **p<0.05; ***p<0.01

Table S2: **Fixed-effect panel regression with robust standard errors.** The model includes dummy variables for the day of the week and season of the year. All independent variables have $VIF \lessapprox 2$, suggesting no multicollinearity.

	New documents
New deaths	0.100*** (0.030)
New deaths (neighbours)	0.190*** (0.040)
Stringency Index	0.35** (0.11)
Vaccinated population % change	1.41*** (0.28)
Observations	22,230
R^2	0.432
Adjusted R^2	0.431

*p<0.1; **p<0.05; ***p<0.01

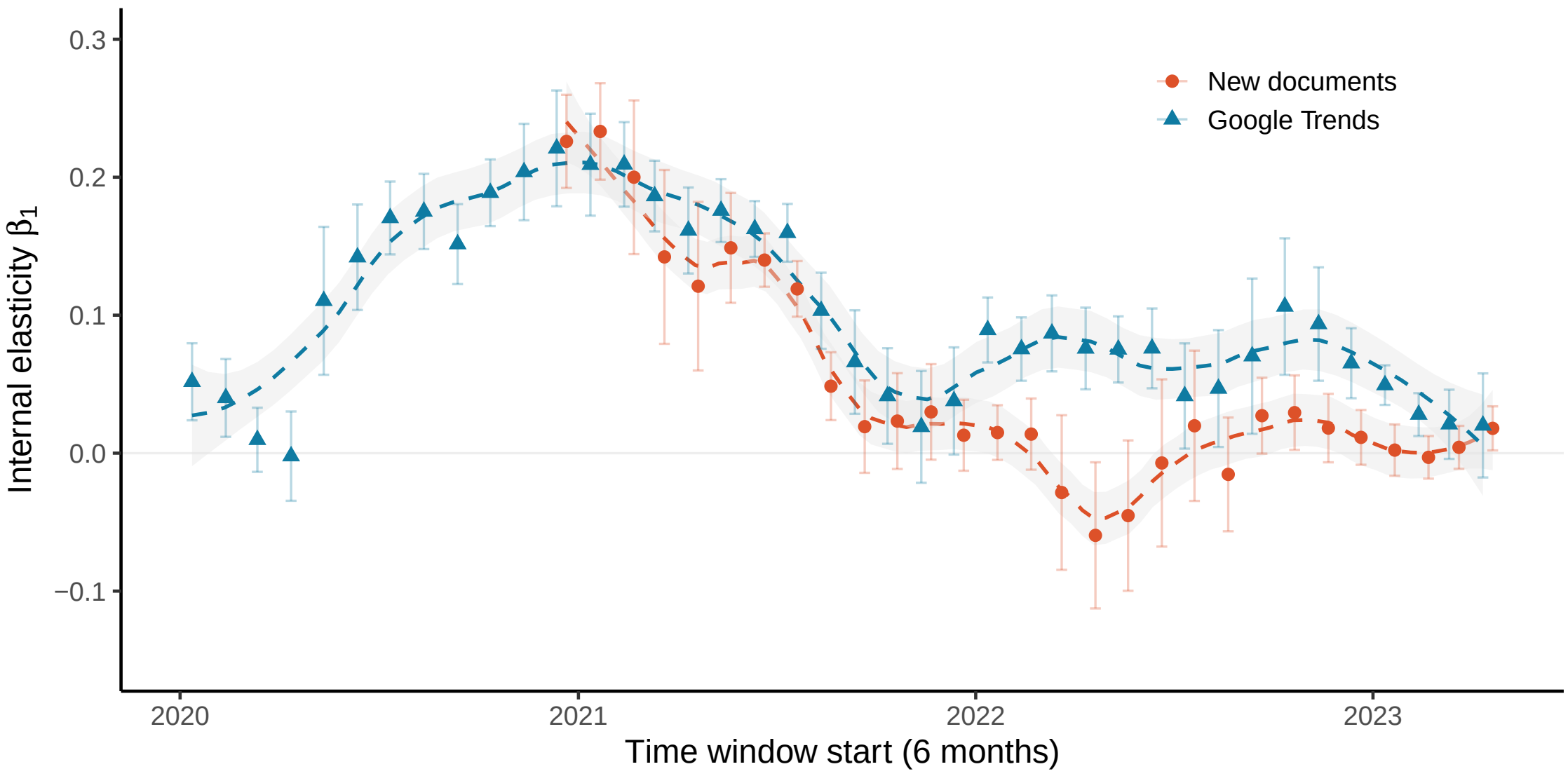

Figure S5: **Internal elasticity associated with the number of new deaths in a panel regression framework.** 'New documents' refers to the data collected by the WHO-EARS platform, whereas 'Google Trends' to the COVID-19 search interest reported by Google.

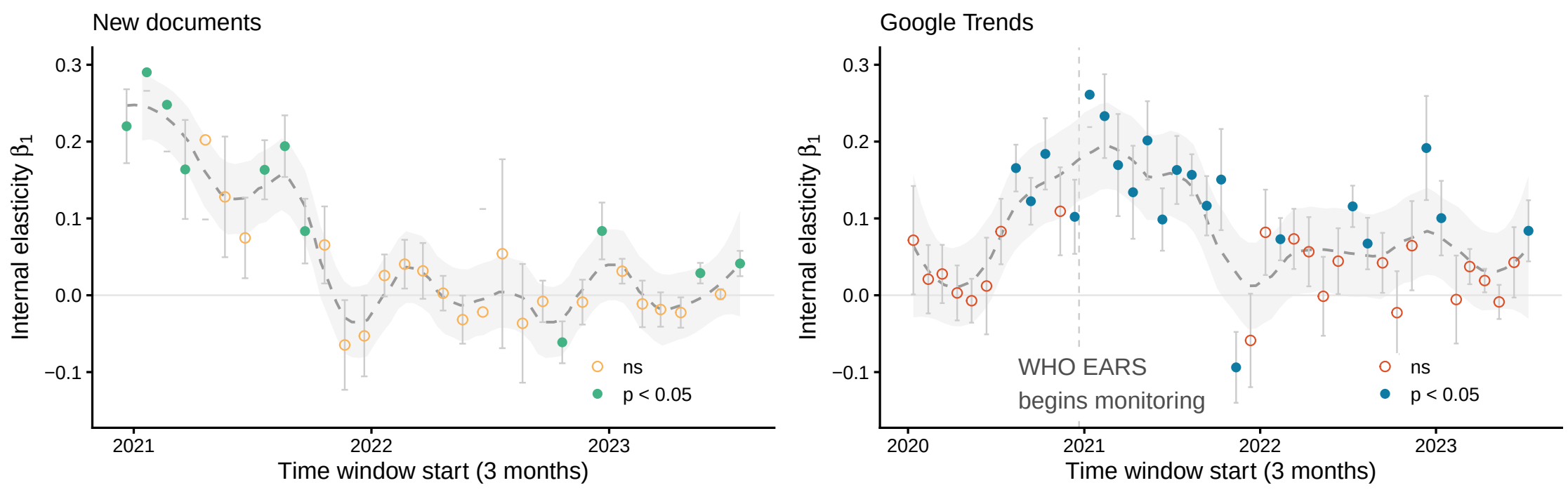

Figure S6: **Internal elasticity associated with the number of new deaths in a panel regression framework.** In the main manuscript, we adopt a rolling window of 6 months, while here we select 3 months as a robustness check. We find broadly consistent results.

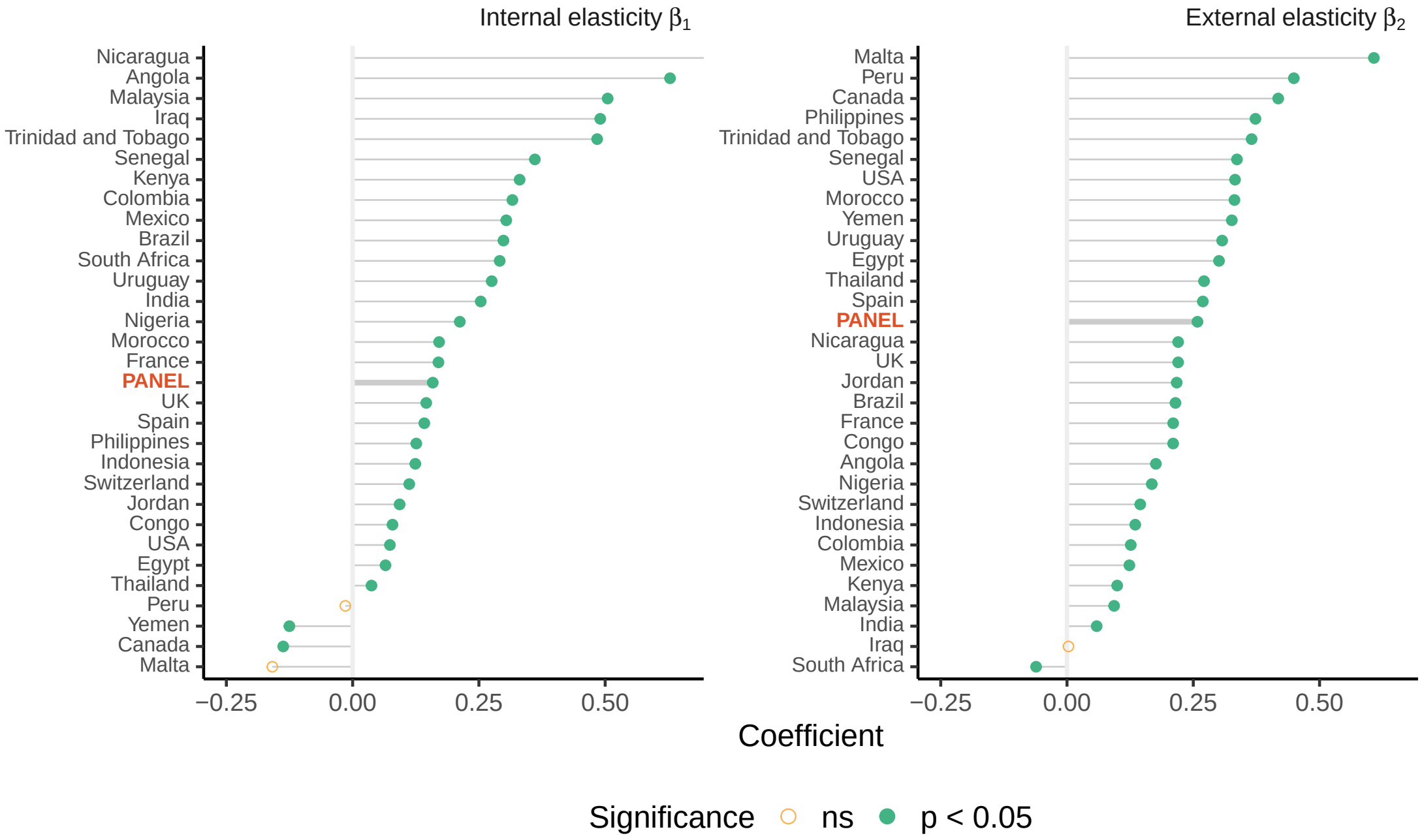

Figure S7: **Internal and external elasticity associated with the number of new deaths in model 1b.** The exact internal elasticity for Nicaragua is omitted from the visualization because much larger than the rest ($\beta_1 \approx 8$).

References

[1] Alejandro Fontal, Menno J Bouma, Adrià San-José, Leonardo López, Mercedes Pascual, and Xavier Rodó. Climatic signatures in the different covid-19 pandemic waves across both hemispheres. *Nature Computational Science*, 1(10):655–665, 2021.

[2] Xavier Rodó and Miquel-Àngel Rodríguez-Arias. A new method to detect transitory signatures and local time/space variability structures in the climate system: the scale-dependent correlation analysis. *Climate Dynamics*, 27:441–458, 2006.

[3] Miquel Àngel Rodríguez-Arias and Xavier Rodó. A primer on the study of transitory dynamics in ecological series using the scale-dependent correlation analysis. *Oecologia*, 138:485–504, 2004.
